# Chromosome 4q25 Variant rs6817105 Bring Sinus Node Dysfunction and Left Atrial Enlargement

**DOI:** 10.1038/s41598-018-32453-8

**Published:** 2018-10-01

**Authors:** Shunsuke Tomomori, Yukiko Nakano, Hidenori Ochi, Yuko Onohara, Akinori Sairaku, Takehito Tokuyama, Chikaaki Motoda, Hiroya Matsumura, Michitaka Amioka, Naoya Hironobe, Yousaku Okubo, Shou Okamura, Hiroshi Kawazoe, Yukie Nishiyama, Hidetoshi Tahara, Kazuaki Chayama, Yasuki Kihara

**Affiliations:** 1 Department of Cardiovascular Medicine, Hiroshima University Graduate School of Biomedical and Health Sciences, Hiroshima, Japan; 2 Department of Internal Medicine, Chuden Hospital, The Chugoku Electric Power Company, Hiroshima, Japan; 3 Department of Gastroenterology and Metabolism, Division of Frontier Medical Science, Programs for Biomedical Research Graduate School of Biomedical Science, Hiroshima University, Hiroshima, Japan; 4 Department of Cellular and Molecular Biology, Graduate School of Biomedical Sciences, Hiroshima University, Hiroshima, Japan

## Abstract

Genome-wide association studies have reported a strong association of the single nucleotide polymorphism (SNP) rs6817105 (T > C) on chromosome 4q25 with atrial fibrillation (AF), but phenotype alterations conferred by this SNP have not been described. We genotyped SNP rs6817105 and examined the relationships among rs6817105 genotype, clinical characteristics, echocardiographic parameters, and electrophysiological parameters in 574 AF patients and 1,554 non-AF controls. Further, multiple microRNAs (miRNAs) are reported to be involved in atrial remodeling and AF pathogenesis, so we investigated relationships between rs6817105 genotype and serum concentrations of 2555 miRNAs. The rs6817105 minor allele frequency was significantly higher in AF patients than non-AF controls (66% vs. 47%, odds ratio 2.12, *p* = 4.9 × 10^−26^). Corrected sinus node recovery time (CSRT) was longer and left atrial volume index (LAVI) was larger in AF patients with the rs6817105 minor allele than patient non-carriers (CSRT: CC 557 ± 315 ms, CT 486 ± 273 ms, TT 447 ± 234 ms, *p = *0.001; LAVI: CC 43.6 ± 12.1, CT 42.4 ± 13.6, TT 39.8 ± 11.6, *p = *0.030). There were no significant differences between rs6817105 genotype and the serum concentrations of miRNAs. These findings strongly implicate rs6817105 minor allele in sinus node dysfunction and left atrial enlargement.

## Introduction

Atrial fibrillation (AF) is one of the most common arrhythmias. Numerous factors, such as hypertension, aging, diabetes, and structural heart diseases, increase AF risk^1^. Genetic background also contributes to AF pathogenesis. Dabar *et al*. reported that at least 5% of all patients with AF and 15% of those with lone AF had a positive family history^2^.

In 2012, Ellinor *et al*. identified six AF susceptibility loci (*PRRX1*, *CAV1*, *C9orf3*, *SYNPO2L*, *SYNE2*, and *HCN4*) in addition to three previously reported loci (*PITX2*, *ZFHX3*, and *KCNN3*) by a genome-wide association study (GWAS) conducted in individuals of European ancestry. More recently, 12 new single nucleotide polymorphisms (SNPs) associated with AF were reported^3,4^. Among them, variants at 4q25, located upstream of *PITX2*, far exceeded the preset threshold for genome-wide significance and demonstrate strong association with AF in both Europeans and Japanese popupations. The *PITX* gene encode the paired-like homeodomain transcription factor family. The mouse *Pitx2* gene encodes three distinct protein isoforms (Pitx2a, b, c), and Pitx2c plays a determinant role in left–right atrium signaling from early somitogenesis onward^5^. It is also reported that human *PITX2* insufficiency results in cellular and molecular changes leading to atrial electrical and structural remodeling linked to arrhythmogenesis^6^.

Many recent reports have implicated various microRNAs (miRNAs) in the electrical and anatomical remodeling associated with AF progression. Lu *et al*. reported that miR-328 contributes to the adverse atrial electrical remodeling in dogs and patients with AF^7^. Cardin *et al*. reported that miR-21 knockdown significantly suppressed left atrial fibrotic remodeling, tissue fibrosis, and AF persistence in rats with heart failure after experimental myocardial infarction^8^.

The mechanisms underlying the association of 4q25/*PITX2* with AF phenotypes have not been elucidated. Here, we investigate the relationships between SNP rs6817105 on chromosome 4q25 identified by previous GWAS^3,4^ and clinical phenotypes of AF, and serum concentrations of 2555 miRNAs.

## Results

### Frequencies of GWAS reported AF associated SNPs in AF patients and non-AF controls

We replicated GWAS reported 9 SNPs (*KCNN3*, *PRRX1*, *CAV1*, *C9orf3*, *HCN4*, *ZFHX3*, *PITX2*, *SYNE2*, *SYNPO2L*)^3^ and compared allele frequencies of these SNPs between AF patients and non-AF controls (Supplementary Table S1). The rs6817105 SNP near *PITX2* was most significantly associated with AF. The rs6817105 minor allele frequency (MAF) was significantly higher in AF patients than non-AF controls (66% vs. 47%, OR 2.12, *p* = 4.9 × 10^−26^).

### Characteristics of AF Patients and Genotypes of rs6817105

The relationships between rs6817105 genotype and various clinical and functional parameters are shown in Table 1. There were no significant differences in clinical characteristics among rs6817105 genotypes in the patient group. However, electrophysiological study revealed a significant linear correlation between rs6817105 and sinus node dysfunction. The maximum sinus node recovery time (SNRT) and corrected SNRT (CSRT) increased with the number of minor C alleles, and were longest in patients with the CC genotype (SNRT: CC 1419 ± 393 ms, CT 1334 ± 342 ms, TT 1275 ± 307 ms, *p* = 0.001; CSRT: CC 557 ± 315 ms, CT 486 ± 273 ms, TT 447 ± 234 ms, *p* = 0.001). Multivariable analysis revealed that rs6817105 genotype and AF type (paroxysmal vs. non-paroxysmal) were independently associated with SNRT >1500 ms (genotype: odds ratio = 1.83, *p* = 0.034; AF type: odds ratio = 2.49, *p* < 0.001). Therefore, sinus node dysfunction was independently associated with the rs6817105 minor allele in these AF patients.

**Table 1:** Characteristics of AF Patients and Genotypes of rs6817105.

Genotype	CC	CT	TT	p
No. of patients	254	244	76	
**Clinical characteristics**				
Age (years)	61.5 ± 9.8	60.9 ± 11.2	64.6 ± 9.7	0.147
Men (%)	198 (78.0)	180 (73.4)	57 (75.0)	0.544
Paroxysmal (%)	157 (61.8)	151 (61.9)	54 (71.1)	0.290
Body mass index (kg/m2)	24.1 ± 3.7	24.1 ± 3.5	23.8 ± 3.5	0.785
Hypertension (%)	146 (57.9)	140 (57.6)	45 (59.2)	0.970
Diabetes (%)	45 (17.8)	34 (13.9)	16 (21.1)	0.484
Ischemic stroke (%)	20 (7.9)	19 (7.8)	2 (2.6)	0.185
Heart failure (%)	6 (2.4)	8 (3.3)	4 (5.3)	0.471
Ischemic heart disease (%)	10 (4.0)	8 (3.4)	2 (2.7)	0.849
**Electrophysiological study**				
AH (ms)	96.5 ± 26.2	96.6 ± 24.3	91.2 ± 24.8	0.237
HV (ms)	42.2 ± 9.9	42.7 ± 28.5	41.8 ± 11.1	0.986
maxSNRT (ms)	1419 ± 393	1334 ± 342	1275 ± 307	0.001
CSRT (ms)	557 ± 315	486 ± 273	447 ± 234	0.001
AERP (ms)	228 ± 37	223 ± 33	224 ± 33	0.323
AVNERP (ms)	315 ± 84	305 ± 77	312 ± 65	0.479
**Echocardiography**				
LAD (mm)	40.2 ± 5.3	39.4 ± 6.3	38.3 ± 6.2	0.019
EF (%)	59.6 ± 7.1	60.0 ± 6.4	60.0 ± 7.5	0.533
LVDd (mm)	48.2 ± 4.2	47.9 ± 5.6	48.8 ± 5.3	0.678
LAV (ml)	75.2 ± 21.0	73.7 ± 24.5	68.4 ± 22.6	0.049
LAVI (ml/cm2)	43.6 ± 12.1	42.4 ± 13.6	39.8 ± 11.6	0.030
LAA area (mm2)	506 ± 170	481 ± 149	473 ± 166	0.055

AERP; atrial effective refractory period, AVNERP; atrioventricular node effective refractory period.

CSRT; corrected sinus node recovery time, EF; ejection fraction, LAA area; left atrial appendage area.

LAD; left atrial diameter, LAV; left atrial volume, LAVI; left atrial volume index,

LVDd; left ventricular diastolic diameter, SNRT; sinus node recovery time.

Echocardiography revealed that left atrial diameter (LAD), left atrial volume (LAV), and left atrial volume index (LAVI) increased with the number of the minor C alleles with largest values in patients with the CC genotype (LAD: CC 40.2 ± 5.3 mm, CT 39.4 ± 6.3 mm, TT 38.3 ± 6.2 mm, *p* = 0.019; LAV: CC 75.2 ± 21.0 ml, CT 73.7 ± 24.5 ml, TT 68.4 ± 22.6 ml, *p* = 0.049; LAVI: CC 43.6 ± 12.1 ml/m^2^, CT 42.4 ± 13.6 ml/m^2^, TT 39.8 ± 11.6 ml/m^2^, *p* = 0.030). Multivariable analysis revealed that rs6817105 genotype and AF type (paroxysmal vs. non-paroxysmal) were independently associated with LAD >40 mm (genotype: odds ratio = 1.78, *p* = 0.029; AF type: odds ratio = 3.02, *p* < 0.001). Thus, left atrium enlargement was independently associated with the rs6817105 minor allele in AF patients.

All other clinical and functional parameters were similar among rs6817105 SNP genotypes in the AF patient group.

### Frequencies of rs6817105 minor alleles in AF patients and non-AF controls before and after matching

Detailed results of rs6817105 genotypes in AF patients and non-AF controls are shown in Table 2. We matched AF patients with controls as for age, gender and BMI using propensity scores matching (Table 3). The standardized difference of the matched samples showed that both AF patients samples and control samples were well balanced. The *PITX2* SNP rs6817105 was still the independent predictor of AF after matching (odds ratio = 3.21, *p* = 3.9 × 10^−14^). Detailed results of rs6817105 genotypes in AF patients and non-AF controls in the matched samples were shown in Table 4. The frequency of MAF was higher in AF patients than that in controls (67% vs 44%, OR 2.52, *p* = 1.0 × 10^−15^**)**. The result was similar before and after propensity score matching.

**Table 2:** The SNP rs6817105 Polymorphism in Patients with AF and non-AF Controls.

						Allelic Model		Dominant Model		Recessive Model	
	Genotype distribution			MAF	HW test	(C vs T)		(CC + CT vs TT)		(CC vs CT + TT)	
	CC	CT	TT		p	p	OR (95% CI)	p	OR (95% CI)	p	OR (95% CI)
AF (N = 574)	254 (44.3%)	244 (42.5%)	76 (13.2%)	0.66	0.15	4.9 × 10^−26^	2.1 (1.8–2.4)	7.3 × 10^−24^	2.8 (2.3–3.4)	5.7 × 10^−12^	2.5 (1.9–3.2)
Control (N = 1554)	344 (22.1%)	782 (50.3%)	428 (27.6%)	0.47	0.71						

The T allele was considered as the reference allele in the allelic model.

The results were tested by the chi-square test and the Cochran–Armitage trend test.

CI; confidence interval, HW; Hardy-Weinberg, MAF; minor allele frequency, OR; odds ratio.

**Table 3:** Univariate Analysis and Multivariate Analysis in Predictors of AF after Matching.

	Original Sample					
	AF	Control	SD*			
N	574	1554				
Age (years)	61 ± 10	38 ± 13	1.795			
Gender (Men)	435 (75.8%)	754 (48.5%)	0.566			
Body mass index (kg/m2)	24.1 ± 3.4	22.2 ± 3.4	0.544			
*PITX2* SNP rs6817105 additive model (CC/CT/TT)	254/244/76	344/782/482	0.520			
	**Matched Sample**					
				**Univariate**	**Multivariate**	
	**AF**	**Controls**	SD*	**P values**	**OR (95%CI)**	**P values**
N	317	317				
Age (years)	57 ± 11	57 ± 11	0.042	6.0 × 10^−1^	1.00 (0.98–1.02)	9.8 × 10^−1^
Gender (Men)	224 (70.7%)	226 (71.3%)	0.014	8.6 × 10^−1^	1.48 (0.69–1.02)	9.3 × 10^−1^
Body mass index (kg/m2)	23.5 ± 3.4	23.9 ± 3.8	0.107	1.8 × 10^−1^	1.09 (0.98–1.04)	1.5 × 10^−1^
*PITX2* SNP rs6817105 additive model (CC/CT/TT)	141/141/35	63/155/99	0.651	1.3 × 10^−15^	3.21 (1.99–2.52)	3.9 × 10^−14^

*SD; Standardized Difference, A standardized difference (of means) <0.25 indicates good balance between groups.

**Table 4:** The SNP rs6817105 Polymorphism in Patients with AF and non-AF Controls after Propensity Score Matching.

	Genotype distribution			MAF	HW test	Allelic Model		Dominant Model		Recessive Model	
						(C vs T)		(CC + CT vs TT)		(CC vs CT + TT)	
	CC	CT	TT		p	p	OR (95% CI)	p	OR (95% CI)	p	OR (95% CI)
AF (N = 317)	141 (42.4%)	141 (46.2%)	35 (11.3%)	0.67	0.97	1.0 × 10^−15^	2.5 (2.0–3.2)	3.3 × 10^−11^	3.2 (2.3–4.6)	4.8 × 10^−10^	3.7 (2.4–5.6)
Control (N = 317)	63 (20.8%)	155 (50.0%)	99 (29.2%)	0.44	0.86						

The T allele was considered as the reference allele in the allelic model.

The results were tested by the chi-square test and the Cochran–Armitage trend test.

CI; confidence interval, HW; Hardy-Weinberg, MAF; minor allele frequency, OR; odds ratio.

### Relationships between rs6817105 and serum miRNA concentrations

Of the 574 AF patients enrolled, serum concentrations of miRNAs were measured in 501. We excluded the 73 patients, because their sample were insufficient. There were no significant differences between *PITX2* SNP rs6817105 genotype and the serum concentrations of miRNAs. We showed the top 50 miRNAs, whose expression levels were higher in patients with the *PITX2* SNP minor C allele than those without (Supplementary Table S2).

## Discussion

In our study, we replicated nine AF-associated SNPs reported in GWAS^3^, in Japanese PAF patients at our institute and confirmed that *PRRX1* SNP rs3903239, *ZFHX3* SNP rs2106261, and *PITX2* SNP rs6817105 were associated with Japanese PAF patients. We focused our attention on 4q25/*PITX2* variant rs6817105, which was most closely associated with AF. Multiple studies have found associations between AF and 4q25/*PITX2* variants^9–11^, but it remains unclear how this SNP affects AF pathogenesis. Here we confirmed the association of *PITX2* SNP rs6817105 with AF in an independent cohort of patients. In our study, the MAF of rs6817105 was 0.47 and it was consistent with the MAF of rs6817105 (0.465) according to phase III HapMap-Japanese data^12^. The association of rs6817105 to AF onset was also confirmed in age, gender and BMI matched AF patients and controls. Furthermore, we reported that the rs6817105 minor allele (C) was independently associated with sinus node dysfunction and enlargement of the left atrium in AF patients. The independence of the associations strongly suggests that the rs6817105 minor allele contributes to structural and electrical remodeling of the left atrium.

Several reports have suggested relationships between atrial remodeling and various miRNAs. MicroRNA-21 levels are increased selectively in fibroblasts of the failing heart^13^.

Overexpression of miR-133 and miR-590 resulted in post-transcriptional repression of TGF-β1 and TGF-βRII, respectively, which reduced collagen production in cultured canine atrial fibroblasts^14^. Levels of miR-146b-5p and miR-155 were independently and positively associated with left atrial dilatation and AF duration^15^. In light of these findings, we examined the relationships between rs6817105 and the serum concentrations of 2555 miRNAs, but there were no significant differences between *PITX2* SNP rs6817105 genotype and the serum concentrations of miRNAs.

Relationship between sinus node function and *PITX2* have been reported in animals. Wang *et al*. reported that *PITX2* positively regulates miR17-92 and miR106b-25, which directly repress Shox2 and Tbx3. Mice with miR17-92 cardio-specific inactivation and miR106b-25 heterozygosity were reported to develop sinus node dysfunction^16^. Chiang *et al*. reported that miR106b-25 cluster binds to ryanodine receptor type 2–3′ UTR and suppresses its translation^17^. In a study by Neco *et al*. increased ryanodine receptor type 2 activity in the sinoatrial node was shown to lead to an unanticipated decrease in its automaticity accompanied by a Ca^2+^-dependent decrease of I Ca _L_ and diastolic SR Ca^2+^ deletion^18^.

The *PITX2*, which is the closest gene to rs6817105, is a critical mediator of left-side morphogenesis. It also represses the sinoatrial node program and pacemaker activity on the left side^5^. Tao *et al*. reported that *PITX2* homozygous mutant mice display variable R-R interval with diminished P-wave amplitude characteristic of sinus node dysfunction^19^. The insufficiency of *PITX2* was reported to result in cellular and molecular changes leading to atrial electrical and structural remodeling linked to arrhythmogenesis^6^. However, Gore-Panter *et al*. reported that chromosome 4q25 variants are not associated with *PITX2* expression in human left atrial appendages (LAA)^20^, the LAA derives from the primordial left atrium (LA)^21^. We queried expression quantitative trait locus (eQTL) data acquired from 264 human left atrial appendage samples available in the Genotype-Tissue Expression (GTEx) website (http://gtexportal.org; V7 release) for cis-eQTL effects of *PITX2* SNP rs6817105. We analyzed genes located within 1 Mb upstream and downstream of *PITX2* SNP rs6817105, but found no genes, including *PITX2*, for which expression was significantly associated with rs6817105. However, these eQTL expression data were not from the target tissue of interest, further investigation is needed to mechanism that *PITX2* SNP rs6817105 modulate sinus node function and LA remodeling.

## Conclusions

We verified that the SNP rs6817105 on chromosome 4q25 is associated with AF, and further show that an increase in the number of minor alleles results in progressively more severe sinus node dysfunction and left atrial enlargement.

## Methods

### Participants

We enrolled 574 Japanese AF patients (435 males and 139 females, mean age 62 ± 10 years) who had undergone radiofrequency catheter ablation at Hiroshima University Hospital from November 2009 to July 2015. We excluded those with severe valvular disease (n = 2), congenital heart disease (n = 2), hypertrophic cardiomyopathy (n = 6), dilated cardiomyopathy (n = 2), and old myocardial infarction (n = 2). We also enrolled 1,554 Japanese non-AF controls (754 males and 800 females, mean age 38 ± 14 years) from Hiroshima University Hospital. The non-AF controls are volunteers collected at the Hiroshima University. Those with cardiac diseases, hyperthyroidism, severe liver and kidney dysfunction and AF were excluded by interview. They may not fully represent the healthy general population because their mean age and the gender ratio differ from those of the AF group as well as the current Japanese population, introducing a potential bias. A propensity score match was also used to clarify the association between this SNP and AF. Age, gender, and BMI were used for matching. The Institutional Ethics Committee of the Graduate School of Biomedical Science at Hiroshima University approved all procedures involving human genome use. Written informed consent was obtained from all participants. All methods were performed in accordance with the relevant guidelines and regulations.

We replicated the GWAS-reported SNP rs6817105 (T > C) on chromosome 4q25^3^ and compared allele frequencies of this SNP between AF subjects and controls. Subsequently, we investigated differences in clinical characteristics, echocardiographic parameters, and electrophysiological parameters, by rs6817105 genotype in AF patients.

We also examined the relationships between the serum concentrations of miRNAs and rs6817105 genotype using serum samples of the AF patients.

### Genotyping

Peripheral blood was obtained from all participants. Genomic DNA was extracted from leukocytes using the QIAamp DNA Blood Mini Kit (QIAGEN, Hilden, Germany) according to the standard protocol. We genotyped *KCNN3* (rs6666258, G > C), *PRRX1* (rs3903239, G > A), *CAV1* (rs3807989, G > A), *C9orf3* (rs10821415, C > A), *HCN4* (rs7164883, A > G), *ZFHX3* (rs2106261, C > T), *PITX2* (rs6817105, T > C), *SYNE2* (rs1152591, G > A), and *SYNPO2L* (rs10824026, A > G) in all participants using the TaqMan assay as previously described^22,23^.

### Echocardiography

Transthoracic echocardiography and transesophageal echocardiography were performed at our institution using a commercially available system (Vivid E9, GE Healthcare, Milwaukee, WI, USA; or iE33, Philips Medical Systems, Andover, MA, USA) before RFCA. Experienced echocardiographers blinded to the genotyping results conducted all echocardiographic examinations and analyzed echocardiographic parameters. Left atrial diameter was measured at end-ventricular systole and left ventricular diameter and interventricular septum thickness were measured at end-ventricular diastole in the parasternal long-axis view. Left ventricular ejection fraction and LAV were measured using the modified Simpson’s formula in the apical four- and two-chamber views^24^. Left atrial volume index was calculated by dividing LAV by body surface area. Left atrial appendage area was measured using transesophageal echocardiography.

### Electrophysiological study

Antiarrhythmic drugs were interrupted 7 days before RFCA. An electrophysiological study was performed during stable sinus rhythm after RFCA using three 5-French-gauge quadripolar electrode catheters, each with a 5-mm inter-electrode distance, positioned at the high right atrium (HRA), His bundle, and right ventricle. The activation intervals of the atrial signal to the His bundle (AH) and the His bundle to the first ventricular signal (HV) were measured on the baseline intracardiac electrocardiography. The SNRT was defined as the recovery interval after a 30-s stimulation from the HRA. The CSRT was defined as the recovery interval in excess of the sinus cycle (i.e., CSRT = maxSNRT − sinus cycle length). The atrial effective refractory period and atrioventricular node effective refractory period were also measured.

### MiRNA

Total RNA was extracted from individual serum samples using the 3D-Gene RNA Extraction Reagent from a liquid sample kit (Toray Industries, Inc., Kanagawa, Japan). A total of 2,555 miRNA sequences were detected using the 3D-Gene miRNA Labeling kit and 3D-Gene Human miRNA Oligo Chip (Toray Industries, Inc). The serum concentrations of miRNAs were globally normalized. We analyzed the relationships between rs6817105 genotype and the serum concentrations of miRNAs in AF patients.

### Propensity Score Matching

Propensity scores were calculated for each patient based on a multivariable logistic regression model. This model included age, sex, and BMI. We matched AF patients with controls using a greedy nearest neighbor method and the overall quality of the matched sample was assessed by comparing the standardized difference of means and the ratio of the variances between the propensity scores of both groups. We evaluated association of the SNP with AF in the matched sample.

### Statistical analysis

Normally distributed continuous variables are presented as mean ± standard deviation. To test genetic associations between cases and controls, we used the χ^2^ test and Cochran–Armitage trend test. Multivariable analysis was performed using a logistic regression analysis. To test the additive genetic effect for the minor allele, the common alleles were coded as 0 (reference), heterozygotes were coded as 1, and minor allele homozygotes were coded as 2. Deviation from Hardy–Weinberg equilibrium was tested among the cases and controls using an ordinary χ^2^ test. Calculations of an each model were as follows, dominant model: comparing genotype AA + Aa vs aa, recessive model: comparing genotype AA vs Aa + aa and allele model: comparing allele A vs a. (Allele A count = (2 × count of AA) + (1 × count of Aa) and Allele a count = (2 × count of aa) + (1 × count of Aa)) Differences among genotypes were analyzed by linear regression for continuous data. Odds ratios (ORs) and 95% confidence intervals are stated as appropriate. A *p*-value of <0.05 was considered significant. We analyzed the relationships between *PITX2* SNP rs6817105 genotype and the serum concentrations of miRNAs, using Bonferroni correction.

## Electronic supplementary material

Supplementary Dataset 1

## Data Availability

The data in this study are available from the corresponding author on reasonable request.
