# Supplementary material for: Chromosome 4q25 Variant rs6817105 Bring Sinus Node Dysfunction and Left Atrial Enlargement
Source: Sci Rep. 2018 Oct 1;8:14565. doi: 10.1038/s41598-018-32453-8 (PMC6167315; doi:10.1038/s41598-018-32453-8)
Supplement: Supplementary file 1 — Supplementary Dataset 1 [file 41598_2018_32453_MOESM1_ESM.pdf]

Chromosome 4q25 Variant rs6817105 Bring Sinus Node Dysfunction and Left Atrial Enlargement

Shunsuke Tomomori, MD[1], [*]Yukiko Nakano, MD, PhD[1], Hidenori Ochi, MD, PhD[2], Yuko Onohara[1] Akinori Sairaku, MD, PhD[1], Takehito Tokuyama, MD, PhD[1], Chikaaki Motoda, MD[1], Hiroya Matsumura, MD[1], Michitaka Amioka, MD[1], Naoya Hironobe, MD[1], Yousaku Ookubo, MD[1], Shou Okamura, MD[1], Hiroshi Kawazoe, MD, PhD[1], Yukie Nishiyama, M.S.[4], Hidetoshi Tahara M.S., PhD[4], Kazuaki Chayama, MD, PhD[3], and Yasuki Kihara, MD, PhD[1]

1.Department of Cardiovascular Medicine, Hiroshima University Graduate School of Biomedical and Health Sciences, Hiroshima, Japan

2.Chuden Hospital, Hiroshima, Japan

3.Department of Gastroenterology and Metabolism, Division of Frontier Medical Science, Programs for Biomedical Research Graduate School of Biomedical Science, Hiroshima University, Hiroshima, Japan

4.Department of Cellular and Molecular Biology, Graduate School of Biomedical Sciences, Hiroshima University, Hiroshima, Japan

Address for correspondence: Yukiko Nakano, MD

Department of Cardiovascular Medicine, Division of Frontier Medical Science, Programs for Biomedical Research

Graduate School of Biomedical Science, Hiroshima University

1-2-3 Kasumi, Minami-ku, Hiroshima 734-8551, Japan

TEL: 81-82-257-5555 (2310)

FAX: 81-82-257-1569

E-mail: nakanoy@hiroshima-u.ac.jp

Supplementary Table S1.

Association of 9 SNPs in AF Patients and non-AF Controls

Resion	SNP-minor allele	Nearest gene	Odds ratio	P value
1q21	rs6666352-C	*KCNN3*	1.34	2.0×10^{-1}
1q24	rs3903239-G	*PRRX1*	1.27	6.0×10^{-4}
7q31	rs3807989-G	*CAV1*	1.11	1.5×10^{-1}
9q22	rs10821415-A	*C9orf3*	1.10	2.3×10^{-1}
15q24	rs7164883-G	*HCN4*	1.04	7.5×10^{-1}
16q22	rs2106261-T	*ZFHX3*	1.55	1.1×10^{-9}
4q25	rs6817105-C	*PITX2*	2.12	4.8×10^{-26}
14q23	rs1152591-A	*SYNE2*	1.14	6.9×10^{-2}
10q22	rs10824026-A	*SYNPO2L*	1.09	2.1×10^{-1}

SNP: single nucleotide polymorphism

Supplementary Table S2.

The association between *PITX2* SNP rs6817105 genotype and microRNA serum concentration (log2)

	PITX2 SNP rs6817105 genotype			
microRNA	CC	CT	TT	p
miR-6768-5p	10.03±1.63	9.81±1.69	9.36±2.25	$7.7×10^{-3}$
miR-6869-5p	13.70±2.31	13.54±2.34	13.09±3.27	$9.9×10^{-2}$
miR-2355-5p	4.06±0.94	3.99±0.96	3.50±1.24	$8.4×10^{-4}$
miR-425-5p	4.43±1.47	4.44±1.36	3.92±1.59	$8.5×10^{-2}$
miR-4508	12.96±2.25	12.83±2.25	12.40±3.01	$1.2×10^{-1}$
miR-151b	5.31±1.54	5.28±1.54	4.81±1.61	$6.3×10^{-2}$
miR-21-5p	5.46±1.57	5.29±1.63	4.89±1.74	$2.8×10^{-2}$
miR-4467	10.87±2.08	11.02±1.65	10.48±2.39	$4.4×10^{-1}$
miR-652-5p	6.06±1.01	5.95±1.09	5.55±1.08	$2.0×10^{-3}$
miR-1973	6.74±1.51	6.69±1.56	6.27±1.26	$6.5×10^{-2}$
miR-3144-5p	3.71±0.97	3.71±1.15	3.27±0.99	$3.9×10^{-2}$
miR-1202	7.99±1.32	7.99±0.99	7.55±1.38	$4.1×10^{-4}$
miR-7114-5p	8.28±1.49	8.06±1.63	7.72±1.70	$1.1×10^{-2}$
miR-3162-5p	7.85±1.10	7.77±1.12	7.36±1.45	$9.0×10^{-3}$
let-7a-5p	5.54±1.72	5.45±1.67	5.06±1.68	$8.5×10^{-2}$
miR-223-3p	5.71±2.03	5.83±1.87	5.33±1.85	$4.1×10^{-1}$
miR-6832-3p	3.90±0.98	3.80±1.01	3.41±1.18	$4.0×10^{-3}$
miR-3907	4.59±1.18	4.40±1.15	4.07±1.43	$4.8×10^{-3}$
miR-2861	12.90±1.77	12.83±1.73	12.44±2.34	$1.2×10^{-1}$
miR-151a-5p	5.35±1.47	5.27±1.44	4.88±1.61	$5.4×10^{-2}$
miR-146b-5p	4.54±1.54	4.52±1.49	4.11±1.64	$1.5×10^{-1}$
miR-6765-3p	7.65±1.64	7.58±1.49	7.20±1.57	$6.9×10^{-2}$
miR-4534	8.85±1.19	8.86±1.32	8.44±1.26	$6.7×10^{-2}$
miR-642a-3p	8.04±1.24	7.86±1.44	7.54±1.40	$8.9×10^{-3}$
miR-221-3p	6.35±1.72	6.45±1.55	5.99±1.56	$3.5×10^{-1}$
miR-361-3p	4.63±1.00	4.44±1.10	4.13±1.19	$1.4×10^{-3}$
miR-4306	4.45±1.19	4.29±1.27	3.97±1.64	$1.4×10^{-2}$
miR-30c-1-3p	6.17±1.10	6.13±1.04	5.75±0.99	$1.7×10^{-2}$
miR-197-5p	8.32±0.68	8.16±1.06	7.85±1.40	$1.0×10^{-3}$
miR-615-3p	4.02±0.92	3.89±0.96	3.56±1.11	$2.2×10^{-3}$

miR-185-5p	4.78±1.36	4.61±1.35	4.31±1.39	2.0×10^{-2}
miR-423-3p	4.66±1.21	4.55±1.18	4.22±1.27	2.3×10^{-2}
miR-1273e	4.33±1.15	4.22±1.13	3.90±1.27	1.6×10^{-2}
miR-4726-3p	4.47±1.96	4.52±2.16	4.12±2.33	4.1×10^{-1}
miR-4485-3p	5.56±1.27	5.47±1.29	5.14±1.13	3.3×10^{-2}
let-7b-5p	5.42±1.74	5.40±1.61	5.03±1.64	2.0×10^{-1}
miR-4459	9.16±0.99	9.02±0.90	8.71±1.29	2.9×10^{-3}
miR-4294	11.39±2.27	11.15±2.42	10.89±2.33	1.0×10^{-2}
miR-5699-5p	4.40±0.94	4.27±1.25	3.96±1.23	9.5×10^{-3}
miR-3131	6.79±0.96	6.7±1.07	6.38±1.11	9.6×10^{-3}
miR-8077	4.52±0.84	4.39±0.93	4.09±0.98	1.1×10^{-3}
miR-3679-5p	7.64±0.97	7.60±1.01	7.25±1.12	2.2×10^{-2}
miR-19b-3p	5.49±1.33	5.32±1.39	5.04±1.41	3.2×10^{-2}
miR-320d	5.12±1.23	5.11±1.23	4.78±1.26	5.1×10^{-2}
miR-6076	6.90±1.24	6.80±1.19	6.49±1.33	2.9×10^{-2}
miR-28-5p	5.06±1.43	5.14±1.30	4.74±1.36	3.3×10^{-1}
miR-6842-5p	7.17±0.93	7.03±0.96	6.74±1.06	2.7×10^{-3}
miR-6740-3p	4.52±1.10	4.37±1.16	4.09±1.19	1.1×10^{-2}
miR-769-3p	3.82±0.89	3.71±0.90	3.41±1.14	7.4×10^{-3}
miR-4481	6.11±1.34	5.95±1.28	5.67±1.30	2.2×10^{-2}
